# Priority indicators for sexual and reproductive health self-care: recommendations from an expert working group

**DOI:** 10.1136/bmjsrh-2023-201909

**Published:** 2023-06-09

**Authors:** Claire W Rothschild, GIlda Sedgh, Martha Brady, Holly McClain Burke, Jane Cover, Andrea Cutherell, Austen El-Osta, Kelsey Holt, Dinesh Kumar, Fredrick Makumbi

**Affiliations:** 1 Department of Sexual and Reproductive Health, Population Services International, Washington, DC, USA; 2 Independent Consultant, Philadelphia, Pennsylvania, USA; 3 Independent Consultant, Washington, DC, USA; 4 Reproductive, Maternal, Newborn, and Child Health Division, FHI 360, Durham, NC, USA; 5 Department of Reproductive Health, PATH, Seattle, WA, USA; 6 Impact for Health, Nairobi, Kenya; 7 Self-Care Academic Research Unit, Imperial College London, London, UK; 8 University of California San Francisco, San Francisco, CA, USA; 9 Dr. R.P. Government Medical College, Himachal Pradesh, India; 10 Department of Epidemiology & Biostatistics, Makerere University, Kampala, Uganda

**Keywords:** family planning services, Health Services Research, HIV, abortion, induced, Reproductive Health

Self-care has been lauded as a strategy to advance universal health coverage by placing users at the centre of health systems, supporting equitable access to health services, and improving health system resilience.^1^ Because self-care is by its nature often practised outside of the formal health system, self-care monitoring and evaluation (M&E) is challenging and requires novel approaches. The WHO has issued and updated global sexual and reproductive health (SRH) self-care guidance,^1^ but M&E standards have not yet been developed. As a result, routine M&E of self-care practice and programmes within national health systems is sparse and fragmented.^2^ Improving the validity, availability, and standardisation of SRH self-care data is critical for strengthening evidence-based self-care delivery models. The Self-Care Trailblazer Group (SCTG) is a global coalition that aims to advance evidence-based SRH self-care policies and programmes. The SCTG Evidence and Learning Working Group (ELWG) led the development of an SRH self-care measurement tool, with the goal of developing a practical and adaptable M&E resource including a set of priority indicators for SRH self-care.

To develop this tool, ELWG recruited a measurement tool development committee comprising 15 members representing academic institutions, non-governmental organisations (NGOs), and clinical practitioners. The committee defined the scope, content, and target users of the tool, and led planning of an expert working group meeting to develop priority indicators. Drawing from extant self-care conceptual models,^3 4^ the committee identified three distinct measurement domains: the enabling regulatory and policy environment; individual knowledge, attitudes, practices, and preferences for SRH self-care; and healthcare service delivery and outcomes. The committee determined that the first edition of the tool would focus on three high-priority SRH self-care interventions that have been the focus of measurement attention and innovation: self-injection of the hormonal contraception subcutaneous depot medroxyprogesterone acetate (DMPA-SC), HIV self-testing, and self-managed abortion.

The committee identified expert speakers, discussants, and participants to engage in a virtual expert working group meeting. Speakers recommended indicators specific to measurement domains and interventions in their areas of expertise, prioritised based on three criteria: usefulness for informing evidence-based decision-making; feasibility of collecting data on the indicator with reasonable and affordable effort; and demonstrated or expected validity of the indictor. More than 70 participants engaged in the 3-day virtual expert working group meeting, held in November 2022, including researchers, implementers, M&E experts, government representatives and donors. Proposed indicators were discussed and debated, with additional feedback from participants captured through online indicator rating polls. After the meeting, speakers convened with the committee to revise and finalise indicators. A consultation session with 13 Kenyan stakeholders representing NGOs, advocates, professional associations, and the Kenyan Ministry of Health was held in January 2023 to capture feedback on the tool’s design and content from target users.

The first edition of the SCTG’s SRH Self-Care Measurement Tool was published online in February 2023 and includes a total of 69 priority indicators.^5^ Each indicator is accompanied by its definition and description of calculations, data sources, and relevant references. A set of illustrative indicators is presented in table 1.

**Table 1:** Illustrative indicators from the SRH Self-Care Measurement Tool, by intervention and measurement domain

Domain	No.	Indicator name	Purpose	Where it’s being used
*DMPA-SC SI*				
Enabling environment	4	Status of policy that authorises private sector staff to initiate SI	Expanding access by reaching women who tend to rely on the private sector for their contraceptive methods	Routine reporting for the DMPA-SC Donor Consortium: Access Collaborative dashboard (https://dashboard.access-collaborative.com); AC country briefs (https://fpoptions.org/resource/ac-country-briefs/)
KAP (knowledge)	12	Percentage of women aged 15–49 who have heard of a self-injectable contraceptive	Gauging knowledge as a precursor for informed decision-making	The Performance Monitoring for Action (PMA) surveys (https://www.pmadata.org/data/survey-methodology)
KAP (practices)	18	Among current DMPA-SC users, percentage of women aged 15–49 who self-injected their current method	Understanding prevalence of SI	The Performance Monitoring for Action (PMA) surveys (https://www.pmadata.org/data/survey-methodology)
Service delivery and health outcomes	20	Number and percentage of service delivery points (SDPs) actively offering SI services	Measuring availability and access, including geographic scope of availability of SI services	Routine reporting for the DMPA-SC Donor Consortium Access Collaborative dashboard & SI quarterly report (https://dashboard.access-collaborative.com)
*HIV self-testing indicators*				
Enabling environment	33	Number of HIVST products approved annually, among those with WHO prequalification	Understanding number of HIVST kits pre-qualified (PQed) or listed by Stringent Regulatory Authority (SRA)/Expert Review Panel for Diagnostics (ERPD).	Collected biannually by WHO
KAP (knowledge)	38	Percentage of HIVST users who would recommend HIVST to a friend	Measuring client satisfaction which covers both their satisfaction with the actual product (ease of use) as well as their satisfaction with the information they received to prepare them to use the product	PSI’s Strengthening HIVST in the Private Sector (SHIPS) project. Data are collected through opt-in chatbot surveys but could also be collected through a more widespread consumer survey
Service delivery and health outcomes	49	Number of people newly enrolled on antiretroviral therapy who report self-test use	Estimating impact of HIVST through use in data triangulation	Usually collected in national DHIS2
*Self-managed abortion*				
Enabling environment	53	National essential medicines list includes combination mifepristone and misoprostol, or misoprostol and mifepristone as separate presentations	Identifying targets to strive for or maintain in area of medicines and other health products	GAPD; IPPF Medical Abortion Commodities Database WHO/HRP multi-country initiative health system monitoring
KAP (knowledge)	56	Percentage of individuals who understand what to expect at each step of the self-managed abortion process	Ensuring quality of abortion care, specifically provision of information to prepare clients	ACQTool, indicator #21 (https://www.acqtool.org/metric/indicators/)
Service delivery and health outcomes	61	Percentage of respondents who reported feeling prepared to determine if their abortion was complete	Facilitating quality SMA requires information and support about abortion completeness. This includes effective communication and preparedness	Field-tested and validated as part of ACQ project ACQTool, indicator #24 (https://www.acqtool.org/metric/indicators/)

AC, Access Collaborative; ACQ, Abortion Care Quality; DHIS2, District Health Information Software; DMPA, subcutaneous depot medroxyprogesterone acetate; GAPD, Global Abortion Policies Database; HIVST, HIV self-testing; HRP, Human Reproduction Programme; IPPF, International Planned Parenthood Federation; KAP, knowledge, attitude, practices; PSI, Population Services International; SI, self-injection; SMA, self-managed abortion; SRH, sexual and reproductive health.

As self-care policies expand around the world, consensus on priority measures and measurement approaches for monitoring self-care is critical. To our knowledge, the SRH Self-Care Measurement Tool presents the first global good for measurement of SRH self-care, and can serve as a practical ‘user guide’ for M&E of SRH self-care for programme implementers and policymakers. It was developed through a replicable process for rapid consensus-driven indicator selection, which may serve as a reference for future efforts to develop standardised indicators across a broader range of self-care interventions.
